# Meis homeobox 2 (MEIS2) inhibits the proliferation and promotes apoptosis of thyroid cancer cell and through the NF-κB signaling pathway

**DOI:** 10.1080/21655979.2021.1923354

**Published:** 2021-05-11

**Authors:** Xiaohui Wen, Mingyu Liu, Jingyan Du, Xun Wang

**Affiliations:** Department of Otolaryngology Head and Neck Surgery, Beijing Chao-Yang Hospital, Capital Medical University, Beijing, China

**Keywords:** MEIS2, thyroid cancer cells, nf-κB signaling pathway, proliferation, apoptosis, p65

## Abstract

Investigation of effects of Meis homeobox 2 (MEIS2) on proliferation and apoptosis of thyroid cancer (TC) cells and its specific molecular mechanism is the main purpose of this study. In this study, we found that the expression of MEIS2 was down-regulated in TC tissues and cell lines (B-CPAP, TPC-1 and K1), compared to adjacent histologically normal tissues and normal thyroid cell (Nthy-ori 3–1). Then, overexpression of MEIS2 promoted B-CPAP cell apoptosis and decreased cell proliferation, viability and cell cycle progression. Further studies confirmed that overexpression of MEIS2 could significantly decrease p65 expression in the nucleus of B-CPAP cells. However, the opposite results were presented after interference of MEIS2 expression. Taken together, MEIS2 expression was significantly down-regulated in TC. In addition, MEIS2 could inhibit NF-κB pathway activation, so as to perform both suppression of the viability and proliferation of TC cells and promotion of apoptosis.

## Graphical Abstract

**Figure:**
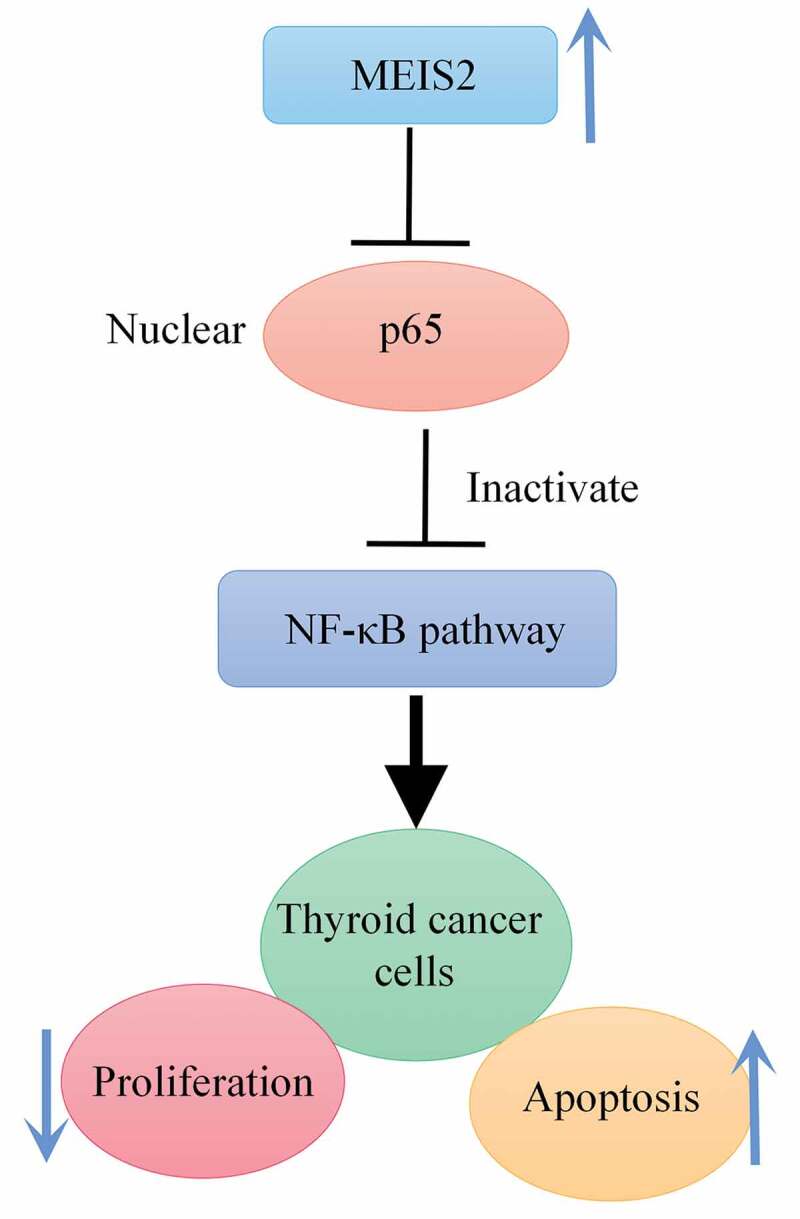

## Introduction

Among malignant tumors of endocrine system positioned in the neck, thyroid cancer (TC) is of a high frequency and its incidence continues to increase. According to the National Cancer Center and epidemiological surveys, 3.4% of cancer patients are new cases of TC each year [1]. The possibility that by 2030, TC is going to be the fourth most common cancer in the world enters into consideration [2]. Based on its pathological features, TC can be categorized into four subtypes (papillary, follicular, anaplastic and medullary) [3], but the papillary-type accounts for 90% to 95% of the total incidence of TC [4,5] and possesses a relative higher malignancy in clinical practice [6,7]. TC affects the safety and quality of life of patients due to its high incidence. Therefore, to find molecular markers has implications for the improvement of clinical diagnosis and treatment of TC.

MEIS family is the family of highly conserved homeobox transcription factor, including Meris homeobox 1 (MEIS1), MEIS2 and MEIS3 [8]. MEIS2 is a branched gene of homeobox genes on chromosome 15q14 [9]. MEIS2 usually forms a protein complex with PBX, which in turn interacts with HOX to promote the transcription of downstream genes and is involved in the processes of ontogeny as well as differentiation [10]. Inhibition of MEIS2 expression has been proved to severely affect the development of the heart [11], brain [12,13], as well as retina [14,15]. Thus, MEIS2 is crucial in organ development. In addition, studies have revealed the correlation between MEIS2 and the occurrence of prostate cancer, ovarian cancer, neuroblastoma and other diseases. For example, the loss of MEIS2 affects the occurrence and progression of prostate cancer and can be used for targeted therapy or for monitoring the condition of a disease [16]. MEIS2 is widely expressed in ovarian cancer and markedly affects its occurrence [17]. And in human neuroblastoma cell lines, MEIS2 is of high expression, and it is the essential requirement for tumor cell proliferation and survival [18]. In addition, Vriens et al. also found that MEIS2 expression was significantly downregulated in patients <40 years old compared to patients with TC >40 years old [19]. However, the exact mechanism of MEIS2 expression in TC has not been reported in the literature.

Therefore, in the present study, we speculated that MEIS2 could act as an oncogene in TC. Based on this, we used a human papillary TC cell line as a study target to investigate its effects and mechanisms on proliferation and apoptosis of thyroid cancer cells by interfering with the expression of MEIS2. In order to provide new targets for the treatment of thyroid cancer.

## 1 Materials and methods

### 1.1 Tissue specimen collection

TC and paracancerous tissues were acquired via TCGA. All tissue samples were diagnosed in the Department of Pathology of the hospital, and all patients did not receive chemotherapy or radiotherapy.

### 1.2 Cell culture and transfection

After acquisition of human normal thyroid cell line Nthy-ori 3–1 and human papillary TC cell lines B-CPAP, TPC-1 and K1 were acquired, RPMI-1640 medium with supplement of 10% FBS and 1% penicillin-streptomycin was used for cell cultures with culture conditions of 37°C, 95% humidity, and 5% CO_2_. According to the transfection kit instructions, B-CPAP cells in logarithmic growth phase were transfected with MEIS2 overexpression vector (MEIS2), negative control vector (NC vector), MEIS2 interference plasmid (sh-MEIS2), and negative control plasmid (sh-NC). Both MEIS2 overexpression and interference plasmids were designed and synthesized by Shanghai Sangon Biological (China).

## 1.3 qRT-PCR

Following the extraction of total cellular RNA by Trizol, the concentration and purity of RNA were determined. After reverse transcription that converting RNA to cDNA under the reverse transcription kit (Thermo, USA) instructions, the cDNA was used to perform the reaction under the qRT-PCR kit instructions. The reaction system was: 95°C for 1 min; 35 cycles of 95°C for 40 s, 58°C for 40 s, and 72°C for 45 s; and 72°C for 10 min. Quantitative analysis using 2^−ΔΔCt^ method was conducted with GAPDH as an internal reference [20].

### 1.4 CCK-8 assay

In 96-well plates, 2000 treated B-CPAP cells were plated in each well added with 100 µL of fresh complete medium. Cell culture was lasted for 24, 48, and 72 hours. Then, each well was supplemented with 10 µL of CCK-8 solution for another 1-hour incubation. Finally, we utilized a microplate reader to measure the optical density (450 nm).

### 1.5 Colony formation assay

Following digestion of the treated B-CPAP cells using 0.25% trypsin, 1640 complete medium containing 0.35% agarose was used to re-suspend the cells. Subsequently, in a 6-well plate, 1 × 10^5^ B-CPAP cells were plated in each well containing 0.6% agarose, followed by incubation at 37°C with 5% CO_2_. The culture was terminated when we could observe colonies with the naked eye. Finally, on completion of staining using 0.1% crystal violet solution, an inverted microscope was utilized to photograph for counting the number of colonies.

### 1.6 Flow cytometry

Annexin V-allophycocyanin Apoptosis Detection Kit (BD Pharmingen, CA, USA) was employed for the identification of cell apoptosis. After the digestion of the cells in each group with trypsin, the B-CPAP cells were centrifuged and then rinsed 2 times with precooled sterile PBS buffer. Subsequently, 1 × 10^6^ cells/ml suspension was prepared with 1× binding buffer. The suspension was gently mixed with Annexin V and nucleic acid dye, and then we kept it out of light at room temperature for a period of 15 min. With addition of 5 µl PI, another 15-min staining was performed avoiding light at room temperature. After that, on completion of adding 400 µl 1× binding buffer, the results were measured within 1 hour using a FACScan flow cytometry system (Becton Dickinson, CA, USA).

### 1.7 Western blot

RIPA lysis buffer was used in the lysing of the cells for 20 min, followed by disruption of the cells using ultrasound in an ice bath. The specific concentration of the collected proteins was worked out using a BCA kit (Beyotime, China). Then after separation using SDS-PAGE, the proteins were blotted onto PVDF membranes, followed by blocking step at room temperature for a period of 1 hour. On completion of the blocking step, overnight incubation of the membrane at 4°C was carried out with primary antibodies MEIS2, p65, GAPDH and Histone (Abcam, UK). After washing the membrane the next day, another 1-hour incubation was carried out with diluted enzyme-labeled secondary antibodies at room temperature. Proteins were developed by a chemiluminescence reagent and placed in a gel imaging system for image acquisition. Protein expression levels were analyzed using GAPDH or Histone as an internal reference.

### 1.8 Statistical analysis

SPSS 25.0 was adopted for statistical analysis. For comparing results between two groups, T test was employed; for comparing among multiple groups, one-way analysis of variance was utilized. Mean ± standard deviation (SD) is the final form to present the outcomes. A statistically significant difference can be suggested if P < 0.05.

## 2 Results

### 2.1 Down-regulation of MEIS2 in thyroid cancer

**Figure 1.**
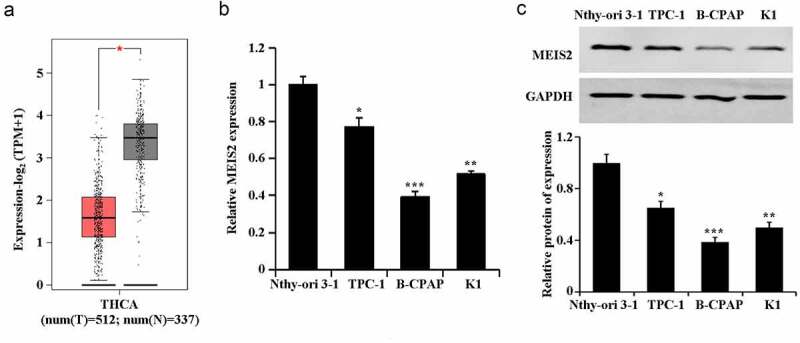
Down-regulation of MEIS2 in thyroid cancer

In the TCGA database, a significant down-regulation of MEIS2 expression was found in TC tissues (Figure 1A). Further, marked decreases of mRNA expression and protein expression of MEIS2 in TC cells were confirmed by qRT-PCR and western blot. Among the TC cells, B-CPAP had the relative lowest expression of MEIS2 mRNA and protein (Figure 1B,C). Therefore, we used B-CPAP cells for subsequent experiments. These results proved the involvement of MEIS2 in TC development.

A. Expression of MEIS2 in thyroid cancer and normal tissues in TCGA database; B. Assessment of MEIS2 mRNA expression in cells using qRT-PCR; C. Evaluation of MEIS2 protein expression in cells using western blot. *P < 0.05, **P < 0.01 and ***P < 0.001 vs. Nthy-ori 3–1 group.

### 2.2 Inhibition of B-CPAP cell proliferation by MEIS2

**Figure 2.**
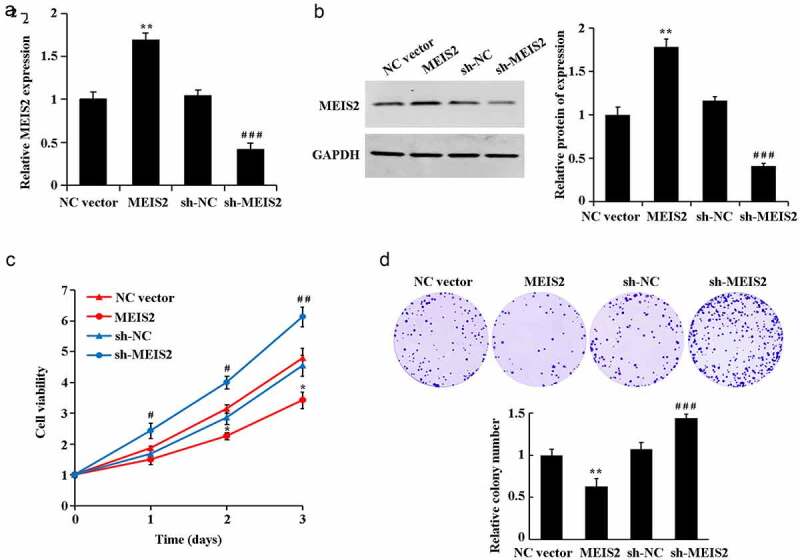
Inhibition of B-CPAP cell proliferation by MEIS2

Further gain- and loss-of-function expression was performed with transfection of MEIS2 overexpression or interference plasmids into B-CPAP cells. qRT-PCR and western blot results were got in determination of transfection efficiency (Figure 2A,B). Compared with the NC vector group, overexpression of MEIS2 induced a significant elevation of cell proliferation rate and cell viability. Compared with the sh-NC group, interference of MEIS2 led to a marked reduction of cell proliferation rate and cell viability (Figure 2C-D, P < 0.05).

A. Determination of MEIS2 mRNA expression in cells using qRT-PCR; B. Assessment of MEIS2 protein expression in cells using western blot; C. Evaluation of cell proliferation utilizing CCK-8; D. Indication of cell viability utilizing colony formation assay. *P < 0.05 and **P < 0.01 vs. NC vector group; ^#^P < 0.05, ^##^P < 0.01 and ^###^P < 0.001 vs. sh-NC group.

### 2.3 MEIS2 promotes apoptosis and inhibits cell cycle progression in B-CPAP cells

**Figure 3.**
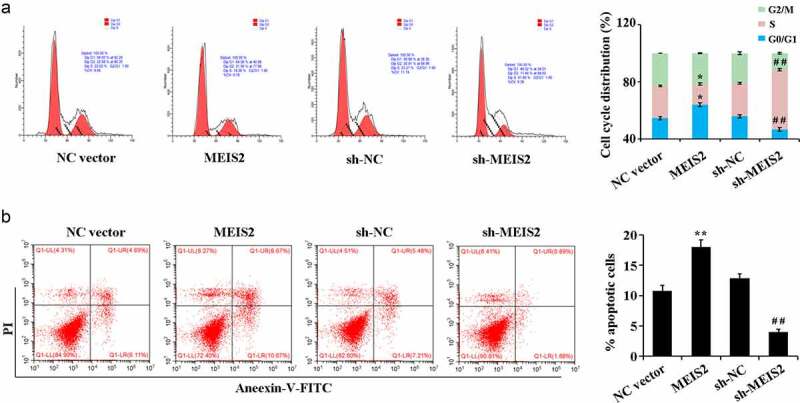
MEIS2 promotes apoptosis and inhibits cell cycle progression in B-CPAP cells

Compared with the NC vector group, overexpression of MEIS2 resulted in marked up-regulation of the apoptotic rate and led to an increase in the number of cells in G0/G1 phase while a decrease in S phase. Compared with the sh-NC group, interference of MEIS2 caused marked down-regulation of the apoptotic rate and led to a decrease in the number of cells in G0/G1 phase while an increase in S phase (Figure 3).

Determination of cell cycle (A) and apoptosis rate (B) by flow cytometry. *P < 0.05 and **P < 0.01 vs. NC vector group; ^#^P < 0.05 and ^##^P < 0.01 vs.sh-NC group.

### 2.4 MEIS2 inhibits NF-κB signaling pathway activity in B-CPAP cells

**Figure 4.**
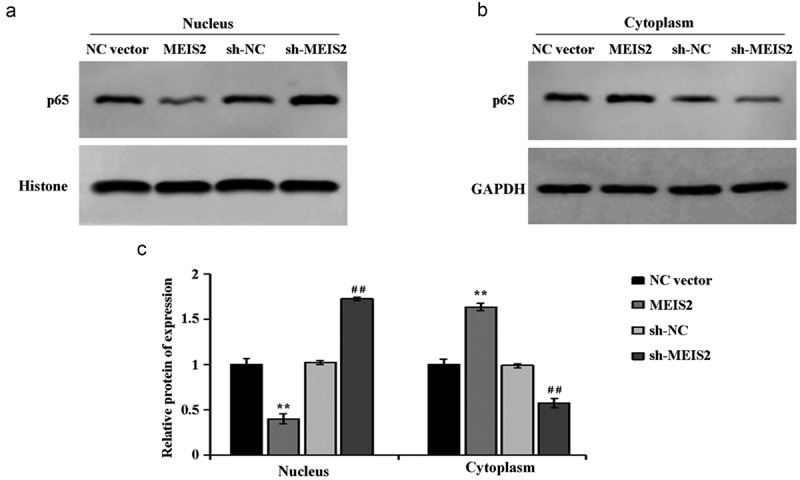
MEIS2 inhibits NF-κB signaling pathway activity in B-CPAP cells

Finally, the molecular mechanism of MEIS2 in suppressing the function of TC cells was investigated. Compared with the NC vector group, in B-CPAP cells, overexpression of MEIS2 induced a down-regulation of p65 expression in the nucleus while an up-regulation in the cytoplasm. However, compared with the sh-NC group, in B-CPAP cells, interference of MEIS2 caused an increase of p65 expression in the nucleus while a decrease in the cytoplasm (Figure 4A,C). Collectively, inhibition of the activity of NF-κB signaling pathway by overexpression of MEIS2 in B-CPAP cells was proved.

Assessment of the expression of p65 in the cytoplasm (A) in and p65 in the nucleus (B) in B-CPAP cells by western blot, and the quantification results (C). **P < 0.01 vs. NC vector group; ^##^P < 0.001 vs. sh-NC group.

## 3 Discussion

TC is considered to be a major contributor to cancer-related death. Asia is the main continent of death cases of TC, while the mortality rate of TC in China ranks second in the world, accounting for about 13.8% [21–24]. Surgical treatment currently occupies the important position in the treatment of thyroid nodules and TC [25]. However, metastasis and recurrence are usually detected after treatment. Therefore, to find new targets for the treatments of TC is of great importance. Zhang et al. found that eIF5A2 was a new prognostic marker and an effective therapeutic target for papillary TC [26].

In the present study, we detected significant down-regulation of MEIS2 in papillary TC tissues and cells, suggesting the involvement of MEIS2 in TC progression. It is also in agreement with the study of Vriens et al. [19]. Further experiments pointed out both inhibition of cell proliferation and viability and promotion of apoptosis and number of cells in G0/G1 phase by overexpression of MEIS2 in TC cells. However, interference of MEIS2 had the opposite effects. These results confirm that MEIS2 inhibits the proliferation of TC cells and promotes their apoptosis.

Furthermore, we investigated the mechanism by which MEIS2 inhibits TC progression. NF-κB, as an important transcription factor, includes p65 (RelaA), RelB, c-Rel, p105/p50 (NF-κB1) and p100/52 (NF-κB2). NF-κB is involved in various reactions and processes, especially in regulating tumor occurrence, proliferation, differentiation, migration and invasion [27–29]. NF-κB will not promote the development of cancer in the early stage of cancer, but play an inhibitory role in cancer. Nevertheless, after enough accumulation of mutations, NF-κB induces gene expression and then promotes tumor occurrence, development and metastasis [30]. The mechanism is that NF-κB in an inactive state binds to IκBα to form a complex presenting in the cytoplasm, and phosphorylation of IκBα leads to ubiquitination and degradation and then releases bound NF-κB-p65. The released NF-κB-p65 is further phosphorylated, followed by enhancement of its nuclear localization signal and entry into the nucleus to promote the expression of downstream genes. Therefore, inhibiting NF-κB-p65 activity is a potential treatment for cancer [31,32]. Our experiments proved the suppression of p65 expression in the nucleus while promotion in the cytoplasm by overexpression MEIS2. And opposite results were got by silencing MEIS2. Based on the above results, it was hypothesized that overexpression of MEIS2 at the cellular level could inhibit p65 protein entry into the nucleus, which in turn inhibited NF-κB signaling pathway activity and ultimately inhibited the process of thyroid carcinogenesis. This provides a new clue for the study of thyroid cancer cell development mechanism. However, the key point and regulatory mechanism of NF-κB signaling pathway in the occurrence and development of papillary TC are required to determine further. Besides, the role of MEIS2 in TC requires further clinical investigation, thus providing new therapeutic methods and therapeutic targets for TC.

## 4 Conclusion

MEIS2 expression is significantly down-regulated in TC. In addition, MEIS2 inhibits the viability and proliferation of TC cells and promoted apoptosis by inhibiting NF-κB-p65. Therefore, MEIS2 can be used as a potential target for early diagnosis as well as targeted therapy in TC patients.

## Supplementary Material

Supplemental Material

## Highlight

1. MEIS2 expression was significantly down-regulated in thyroid cancer.
2. MEIS2 could inhibit thyroid cancer cell proliferation and promote apoptosis.
3. MEIS2 could inhibit NF-κB pathway activation.
