# Supplementary figures and images for: Meis homeobox 2 (MEIS2) inhibits the proliferation and promotes apoptosis of thyroid cancer cell and through the NF-κB signaling pathway
Source: Bioengineered. 2021 May 11;12(1):1766–72. doi: 10.1080/21655979.2021.1923354 (PMC8806259; doi:10.1080/21655979.2021.1923354)

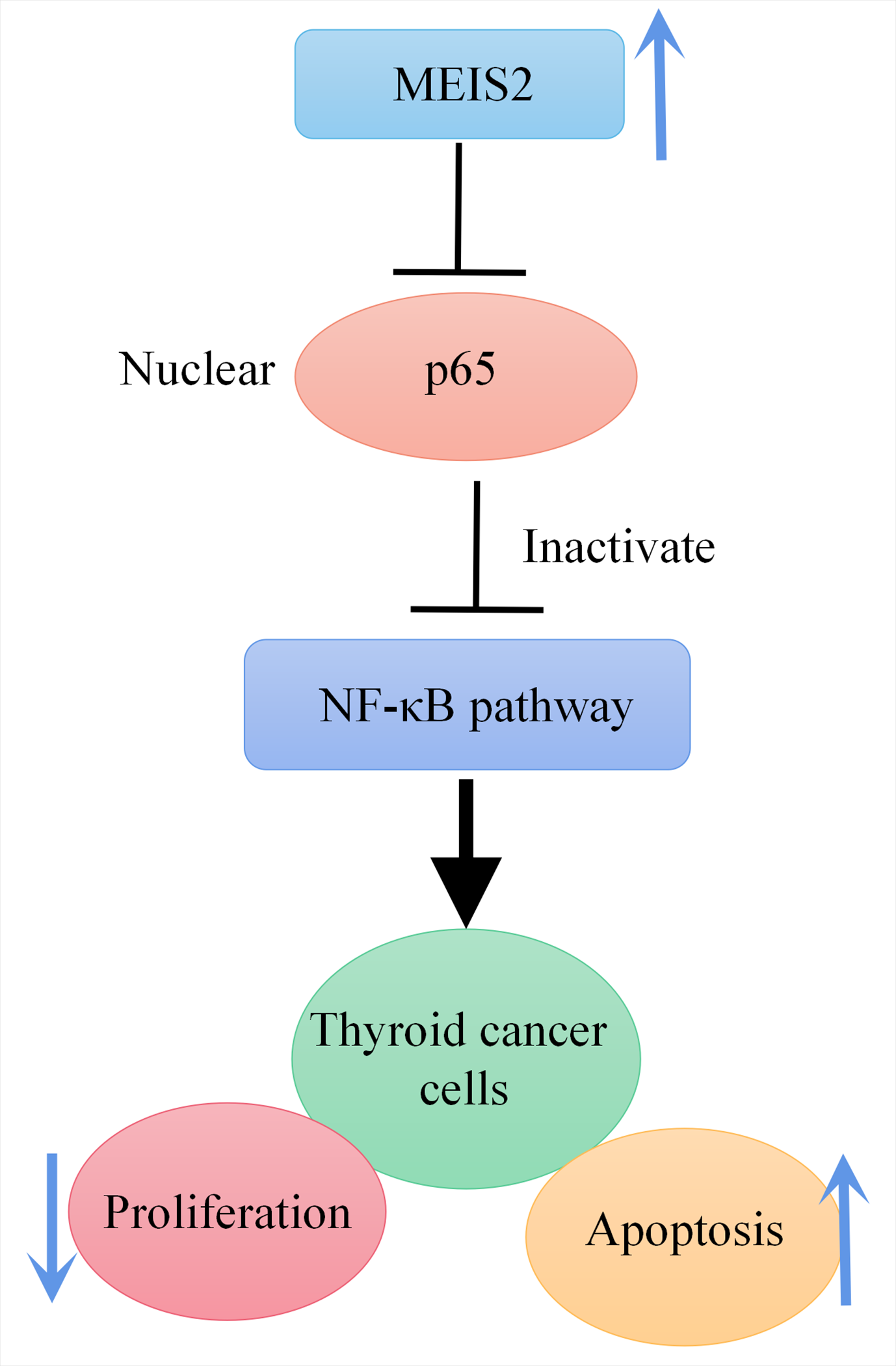

Supplement: Supplemental Material [file KBIE_A_1923354_SM9995.tif]
